# Durable, transparent and superhydrophobic coating with temperature-controlled dual-scale roughness by self-assembled raspberry nanoparticles

**DOI:** 10.1016/j.heliyon.2024.e34983

**Published:** 2024-07-23

**Authors:** Brahim Nomeir, Sara Lakhouil, Sanae Naamane, Mustapha Ait Ali, Sofia Boukheir

**Affiliations:** a Molecular Chemistry Laboratory, Unit Coordination and Catalysis Chemistry, Cadi Ayyad University, Faculty of Sciences Semlalia (UCA-FSSM), B.P. 2390, 40000, Marrakech, Morocco; b Moroccan Foundation for Advanced Science Innovation and Research (MAScIR), Rue Mohamed Al Jazouli, Rabat, Morocco; c Mohammed V University, Mohammadia School of Engineers (EMI), Avenue Ibn Sina B.P. 765 Agdal, Rabat, Morocco

**Keywords:** Superhydrophobic, Self-cleaning, Coating, Transparency, Durability

## Abstract

This study focuses on creating a superhydrophobic, durable, and exceptionally transparent coating with dual-scale roughness by naturally formed raspberry-like particles. This approach facilitates the management of surface roughness at both single and dual scales through variations in surface functionalization temperature. We illustrated that adjusting the temperature of organosilanes functionalization on the surface allows for various reactions, such as the direct grafting of metallic precursors or their polymerization on the surface, resulting in the formation of large raspberry-like particles.

We investigated the impact of nanoparticle concentration, functionalization duration, and reaction temperature on surface properties. Our results reveal that a concentration of 1.5 % SiO_2_ nanoparticles, combined with surface functionalization using TCMS for 4 h at 3 °C, provides the optimal conditions for creating a surface that combines superhydrophobicity, transparency, and acceptable durability. The resulting surface exhibits an impressive contact angle of 158.9°, a sliding angle of 2°, and a transmittance rate of 82 %.

Furthermore, the coating demonstrates remarkable resistance to abrasion for up to 35 cycles and can withstand temperatures up to 280 °C. It also offers enhanced protection against UV radiation for 50 h and improved resistance to sand abrasion for up to 30 s, enduring bombardment pressures of up to 6 bars. Moreover, the coating presents several advantages in terms of surface cleaning.

## Graphical abstract

**Figure:**
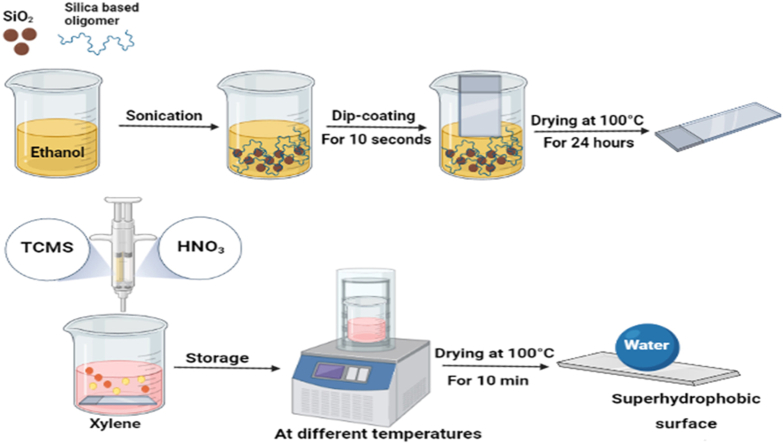

## 1 Introduction

The development of transparent superhydrophobic coatings with satisfactory durability is a crucial subject for both academic research and industrial applications. Transparent superhydrophobic surfaces are defined by a contact angle (CA) exceeding 150°, a sliding angle (SA) below 10°, and a transparency surpassing 80 % [1,2]. These characteristics enable water droplets on the surface to assume a spherical shape and roll off easily with slight disruption, effectively removing dust and making the surface self-cleaning [3,4].

Significant efforts have been dedicated to creating advanced synthetic superhydrophobic coatings with self-cleaning, dirt-repellent, and anti-corrosion properties [[5], [6], [7]]. These coatings find diverse applications, including glazing, solar cells, lenses, and windshields [8,9]. However, manufacturing superhydrophobic coatings with high transmittance poses a challenge due to the inherent trade-off between superhydrophobicity and transparency. The hierarchical micro/nano-structures required to achieve superhydrophobicity can induce notable light scattering, thus diminishing transparency [10,11].

As always, nature serves as a source of inspiration in addressing our challenges. Drawing inspiration from the mosquito's eyes, a promising new approach has been discovered for designing transparent superhydrophobic coatings [1,12]. Typically, this approach involves fabricating coatings with surface structures smaller than the wavelength of visible light to minimize light scattering. These surface structures are modified using materials with low surface energy [1].

However, despite the fabrication of various transparent superhydrophobic coatings, their practical applications remain confined to the laboratory scale due to their limited lifespan [13,14]. Low surface energy materials degrade rapidly under the influence of various factors, including UV rays, temperature, abrasion, and the presence of oxidizing substances in the air or water. Consequently, the development of a coating that combines all three properties—superhydrophobicity, transparency, and durability—is an essential area of interest for both industrial applications and academic research.

The use of nanoparticles of different sizes or the use of raspberry nanoparticles is considered one of the tricks used to solve this issue. This technique significantly improves surface roughness compared to using single-sized nanoparticles. This helps enhance surface roughness without causing a significant impact on transparency [[15], [16], [17]]. Zhao et al. [18] prepared a superhydrophobic and highly transparent coating by utilizing a mixture of double-sized silica particles (55/200 nm) and epoxy resin to enhance the adhesion of the particle films. Additionally, Zhi et al. [19] introduced a method based on the creation of a dual-scale structure. The coating synthesized using this approach exhibited remarkable properties, including excellent hydrophobicity (CA/SA of 157.9°/1.2°), high transparency (average transmittance of 97 %), and a refractive index of 1.3471. In a separate study, Yan et al. [20] successfully developed an anti-wetting and extremely transparent surface by incorporating raspberry nanoparticles on various substrates, such as glass and polyvinyl chloride (PVC). The coated surfaces demonstrated a 2 % increase in transparency compared to the uncoated substrates.

On the other hand, the use of polymeric resin in combination with nanoparticles can be a very interesting solution to enhance the lifespan of coatings [[21], [22], [23]]. However, so far, we have not yet achieved acceptable durability on an industrial scale. Only a few resins demonstrate significant durability, such as PDMS [24,25], but this material is too expensive, limiting its application and making it economically unviable for large-scale industrial use. To address this issue, the use of inorganic silica-based resins prepared via the sol-gel method can be a better solution [26,27]. Firstly, they offer extremely high transparency and improved compatibility with glass substrates due to their similar chemical nature. They adhere to glass substrates through covalent bonds, significantly enhancing their mechanical stability. Moreover, the sol-gel process has proven to be an effective, simple, and cost-effective strategy compared to other techniques.

Raspberry nanoparticles have attracted the attention of researchers for the preparation of highly hydrophobic coatings. The presence of numerous wetting stabilization points on the surface results from the hierarchical roughness inherent to the distinct morphology of the raspberry bush. Within raspberry nanoparticles, the integrated hierarchical topography facilitates the enhancement of secure physical roughness, thereby enhancing superhydrophobicity. This resilience is particularly advantageous because it reduces susceptibility to structural disruptions caused by nanoparticle aggregations during coating processes such as spraying. Xia et al. [17] proposed a method to prepare a superhydrophobic coating based on raspberry-like nanoparticles in three steps. To obtain these nanoparticles, they first prepared large nanoparticles based on styrene-acrylic acid copolymer, then TEOS was polymerized on the surface of these nanoparticles, forming small particles attached to the copolymer-based particles, thus achieving the final hierarchical raspberry-like particles. After functionalizing these raspberry-like nanoparticles, they were deposited on the surface, exhibiting a contact angle greater than 150° and a sliding angle less than 10°. In another study, Kim et al. [28] prepared a superhydrophobic surface using the spray method, depositing a coating composed of both raspberry-like and homogeneous polystyrene nanoparticles. To achieve this, they added styrene/xylene/divinylbenzene dropwise onto a P123/DI solution. The solution was degassed, then an initiator was added to initiate polymerization. Finally, raspberry-like nanoparticles were obtained after washing and centrifugation of the solution. The coating exhibited a contact angle greater than 150°. Mamata [29] reported a method for preparing a transparent self-cleaning coating based on silica and titanium nanoparticles with a raspberry-like structure, due to the in situ generation of particles. For the preparation of the raspberry nanoparticle formulation, they employed a complex process involving the use of additives, dispersants, resin, as well as acids. Li et al. [30] worked on the fabrication of a superhydrophobic coating based on raspberry-like nanoparticles using the dip-coating technique. Initially, they prepared small epoxy-functionalized SiO_2_ nanoparticles and then deposited them onto large polydopamine nanoparticles to create hierarchical raspberry-like nanoparticles. Subsequently, the functionalized surfaces were immersed in the coating. Generally, their preparation involves grafting, polymerization or depositing small particles onto larger ones in suspension to achieve the raspberry structure, followed by coating the substrate with the suspension. The surface exhibited a contact angle of 156° with very good chemical and thermal stability. Based on these studies, the fabrication of superhydrophobic coatings using raspberry-like nanoparticles holds promise; however, it requires a complicated, multi-step process and the use of several ingredients.

In this study, we propose for the first time, a highly simple method for the first time to prepare a superhydrophobic and hyper-transparent coating via the sol-gel method assisted by dip-coating, with a dual-scale roughness utilizing self-assembled raspberry-like nanoparticle structures. Our approach aims to ensure that raspberry-shaped nanoparticles are self-formed on the surface without needing to synthesize these raspberry nanoparticles in suspension and then deposit them on the surface subsequently. Initially, SiO_2_ nanoparticles were prepared using the sol-gel method, involving the combination of 60 nm-sized SiO_2_ nanoparticles with an inorganic resin, followed by the random polymerization of methyltrichlorosilanes on the nanoparticle surface. This step creates large raspberry-like nanoparticles, enhancing surface roughness and achieving dual-scale roughness while maintaining glass transparency. The results demonstrated a contact angle of 158.9°, a sliding angle close to 2°, and a transmittance of 82 %. Self-cleaning tests were conducted on the coated surface, exhibiting excellent performance. Furthermore, the coating exhibited exceptional chemical, mechanical, and thermal stability, along with improved resistance to UV radiation. This method opens new prospects for designing highly functional surfaces for various industrial applications.

## 2 Experimental

### 2.1 Materials

Glass substrate of 3 cm thickness purchased from a local store, Ammonia (NH_4_OH), Trichloro (methyl)silane (TCMS), absolute Ethanol (EtOH), and Tetraethylorthosilicate (TEOS), Xylene and Nitric acid (HNO_3_) were provided by Sigma Aldrich. All the chemicals were used as received without further purification.

### 2.2 Preparation of superhydrophobic coatings

The Stöber method was used to create SiO_2_ nanoparticles with a size of 60 nm. In this process, 5 mL of TEOS were dissolved in 38.53 mL of ethanol, followed by the dropwise addition of 0.4 mL of ultra-pure water and 3 mL of ammonium hydroxide (NH_4_OH). The resulting mixture was stirred at 60 °C for 1 h. Then, the product was centrifuged and washed several times with ethanol. The supernatant was discarded, and the resulting precipitate was dried at 80 °C to remove the solvent and obtain the SiO_2_ powder. The size of the obtained nanoparticles was confirmed by Zetasizer and SEM analysis (see supplementary file).

After the preparation of the nanoparticles, the coating formulation was carried out using the sol-gel method. In a flask, demineralized water was added, and hydrochloric acid was gradually added until reaching a pH of 2. This is because silicic acid exhibits its highest temporary stability in the absence of salt, and the gelation process takes longer within a pH range of 1.5–3. Subsequently, 15 mL of this solution were mixed with 10 mL of TEOS in a round-bottom flask and stirred at room temperature for 2 h, resulting in the formation of the Si(OH)_4_ sol.

During this period, varying amounts of SiO_2_ nanoparticles ranging from 0.5 % to 2 % were dispersed in 10 mL of ethanol and subjected to 1 h of sonication to form stable SiO_2_ suspensions. Subsequently, the SiO_2_ suspensions were mixed with Si(OH)_4_ to obtain a 1 % volume-to-volume (v/v) solution, and the reaction mixture was stirred and sonicated for 1 h at a temperature of 60 °C to create covalent bonds between the resin and the nanoparticles.

To ensure the quality of the prepared coatings, a preliminary step of thorough cleaning of the glass substrates was carried out using an ultrasonic bath for 15 min to remove impurities. This process was performed sequentially using water, ethanol, and acetone. Subsequently, the substrates were dried in a 75 °C oven for 1 h.

**Fig. 1:**
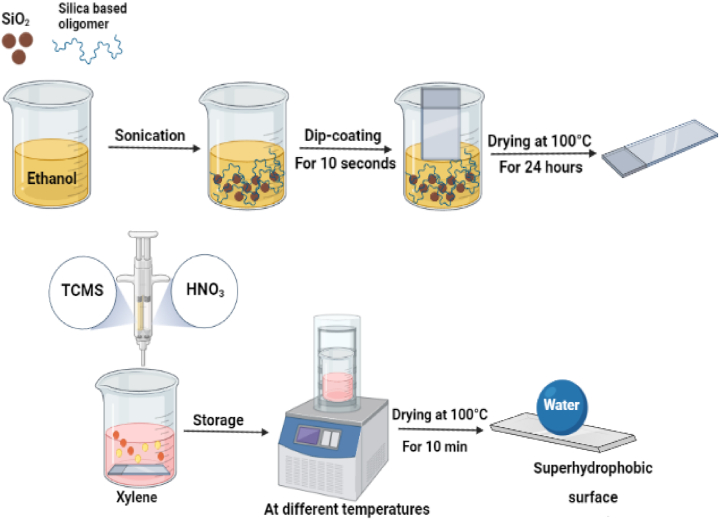
Schematic illustration of the preparation of the transparent superhydrophobic coating.

Once the substrates were ready, they were immersed in the coating suspensions for 10 s and then carefully withdrawn. These coated substrates were then dried for 24 h in an oven at a temperature of 100 °C. This process established bonds between the resin and the substrate, as well as between the resin and the nanoparticles, through a dehydration reaction. This reaction transformed the hydrogen bonds between Si–OH groups into Si–O–Si covalent bonds, ensuring a strong adhesion of the coating [31]. Next, the coated glass slides were immersed in a beaker containing 50 mL of xylene. To complete the process, 500 μL of methyltrichlorosilane were added to the beaker, followed by the addition of 1.25 mL of nitric acid. The mixture was kept at different temperatures (60 °C, 25 °C, 10 °C, and 3 °C) for varying durations ranging from 2 to 6 h. Once the reaction was completed, the coated substrates were rinsed three times with xylene and ethanol, then dried for 10 min at 100 °C to finalize the coating process (Fig. 1).

The functionalization of nanoparticles was analyzed using various techniques. Total analysis attenuated Fourier transform infrared spectroscopy (ATR-FTIR) was employed to study the chemical composition. The samples were examined using a Nicolet iS5 FTIR spectrometer. Additionally, surface morphologies were observed using a scanning electron microscope (SEM, SUPRA55) with an accelerating voltage of 5.0 kV. Before analysis, the samples were coated with a thin layer of gold. The coating thickness was measured using profilometry (Bruker XP-1 Model, Germany). The surface transmittance was studied in the visible range using a Cary 5000 UV–Vis spectrophotometer. To determine the contact angle and slip angle of the superhydrophobic surfaces, a goniometer was used. Deionized water droplets were extruded from a needle at room temperature, and if the water drop did not adhere to the surface, the contact angle and slip angle values were obtained by measuring at least 5 different points on each surface. Atomic force microscopy (AFM) in non-contact mode, utilizing a Veeco Dimension icon instrument, was used for three-dimensional film imaging and surface roughness measurements. The AFM image analysis was performed using NanoScope-Analysis-v110R1 software.

For each sample, we performed five measurements per sample for each metric and each durability test. The error bars were calculated as the standard deviation of these measurements. The detailed method for calculating these error bars is included in supplemantary file.

## 3 Results and discussion

### 3.1 The impact of temperature on surface morphology and wettability

**Fig. 2:**
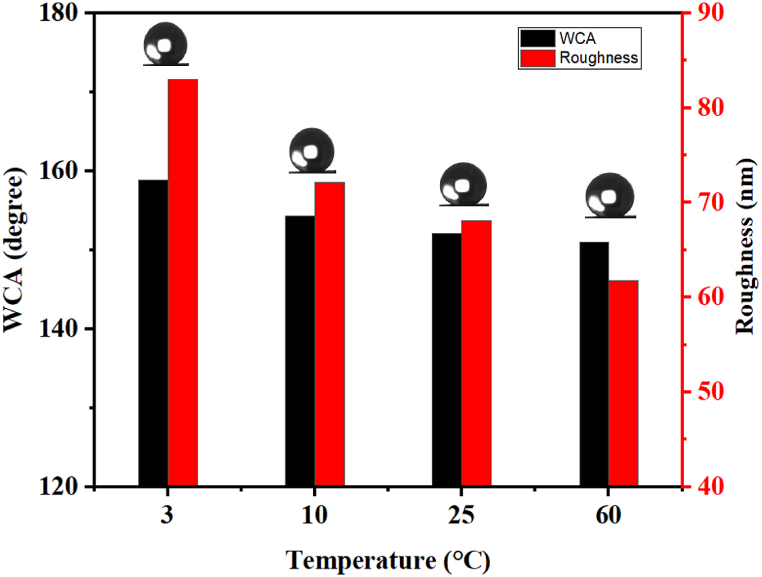
The variation of the WCA contact angle and roughness as a function of temperature variations during the functionalization reaction.

The reduction of surface energy is an essential requirement to achieve superhydrophobicity according to the Cassie-Baxter model [32]. For this purpose, after the deposition of nanoparticles, it is necessary to functionalize the surface with low surface energy molecules to hinder the surface's ability to form bonds with water droplets and promote droplet sliding, thereby inhibiting adhesion. However, it is evident from the literature that most research studies have employed relatively high temperatures for organosilane grafting on coated surfaces [33]. To the best of our knowledge, we are the first to investigate the temperature's effect on the functionalization of organosilane-based coatings. In this investigation, we maintained a reaction period of 4 h and a SiO_2_ nanoparticle concentration of 1.5 %. Four distinct temperatures, namely 3 °C, 25 °C, 10 °C, and 60 °C, were used for the process. The contact angle results are summarized in Fig. 2. When the reaction was carried out at 60 °C, the surface exhibited a contact angle of 151°. However, at 25 °C, the contact angle measured 152.1°, and at 10 °C, the surface showed a contact angle of 154.3°. Interestingly, an angle of contact of 158.9° was obtained when the reaction was conducted at 3 °C, highlighting the significant impact of reaction temperature on the contact angle. This shows that carrying out functionalization reactions using this low-temperature approach can significantly enhance superhydrophobicity, compared to high temperatures or room temperature, which can bring a benefit for several works, as the works of Zhang et al. [26], Chong et al. [13], and others [34,35].

Furthermore, AFM was employed to evaluate the surface roughness of the three samples (see Fig. 2). It was demonstrated that surface roughness increases as the reaction temperature decreases even if the nanoparticle concentration is the same, and this correlation holds true for the contact angle measurements.

**Fig. 3:**
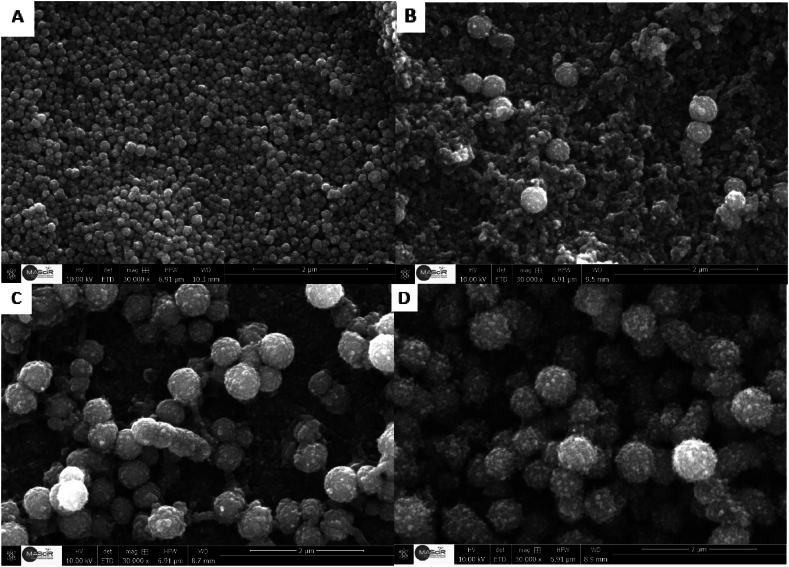
SEM images of the surface morphology after the deposition of the coating according to the different temperatures: A) at 60 °C, B) at 25 °C, C) at 10 °C and D) at 3 °C.

The surfaces were examined by SEM to determine how temperature affected surface roughness and contact angle, and the results are presented in Fig. 3. When the functionalization reaction is performed at 60 °C, we can clearly see that the nanoparticles are of the same size and well-distributed on the surface. However, when the reaction was conducted at 25 °C, the formation of significantly larger nanoparticles, considerably larger than the SiO_2_ nanoparticles, was observed. At 10 °C, the larger nanoparticles, on the other hand, became more visible and started to resemble raspberries. When the reaction was carried out at 3 °C, the surface was completely covered by these larger raspberry-like nanoparticles. This resulted in a dual-scale roughness, making the surface more hierarchical and increasing the surface roughness accordingly. This enables the regulation of surface roughness at a single or dual scale by temperature fluctuations during functionalization.

**Fig. 4:**
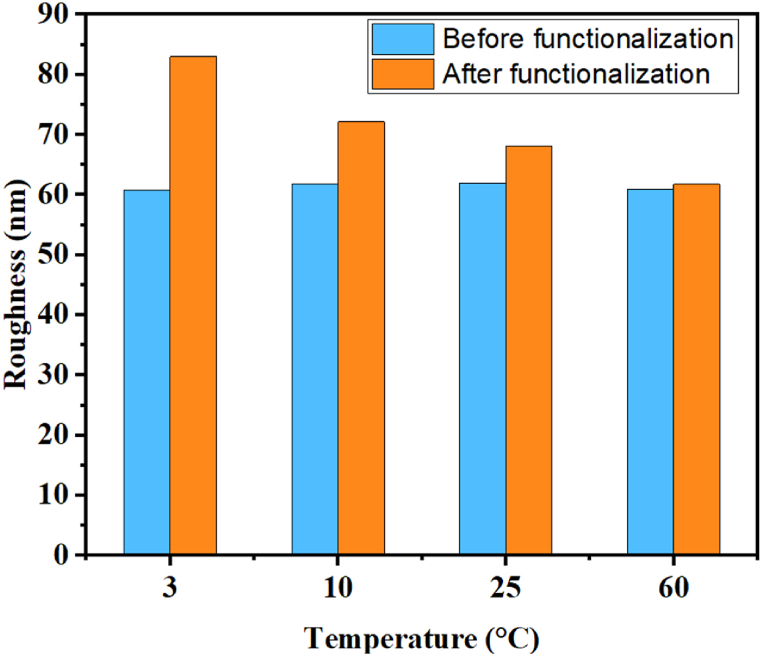
Surface roughness before and after surface functionalization at different temperatures.

**Scheme 1:**
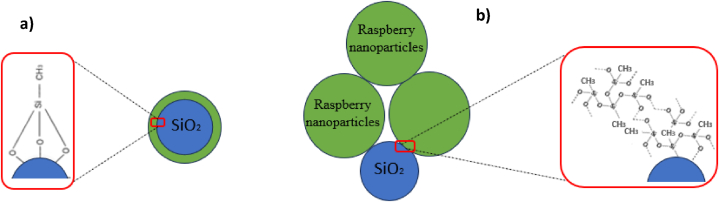
Schematic explanation of the resulting reactions as a function of the functionalization temperature: a) high temperatures, b) low temperatures.

This can be explained by the type of reaction occurring in the reaction medium. As shown in Fig. 2, Fig. 3. After functionalization at 60 °C, we obtained superhydrophobicity, and the surface showed that SiO_2_ nanoparticles of very small size and in Fig. 4 we can notice the surface roughness is not changed too much After functionalization. which shows that apparently, at a temperature of 60 °C, after the hydrolysis of TCMS, each precursor binds to the 3 OH groups of the SiO_2_ nanoparticles, forming three covalent bonds. This allows the grafting of TCMS without altering the morphology of the nanoparticles or the surface roughness. In contrast, during functionalization at a temperature of 3 °C, we achieved superhydrophobicity, but the roughness increased by 23 nm (Fig. 4) with the appearance of new hydrophobic raspberry-like nanoparticles. This indicates that at a temperature of 3 °C, after the hydrolysis of TMCS, the reaction tends to target the surface of the nanoparticles with one or two groups, while the remaining groups participate in an inorganic polymerization reaction with other TCMS precursors (Scheme 1). Consequently, large raspberry-like nanoparticles form alongside the SiO_2_ nanoparticles, resulting in the creation of a self-assembled dual-scale roughness.

**Fig. 5:**
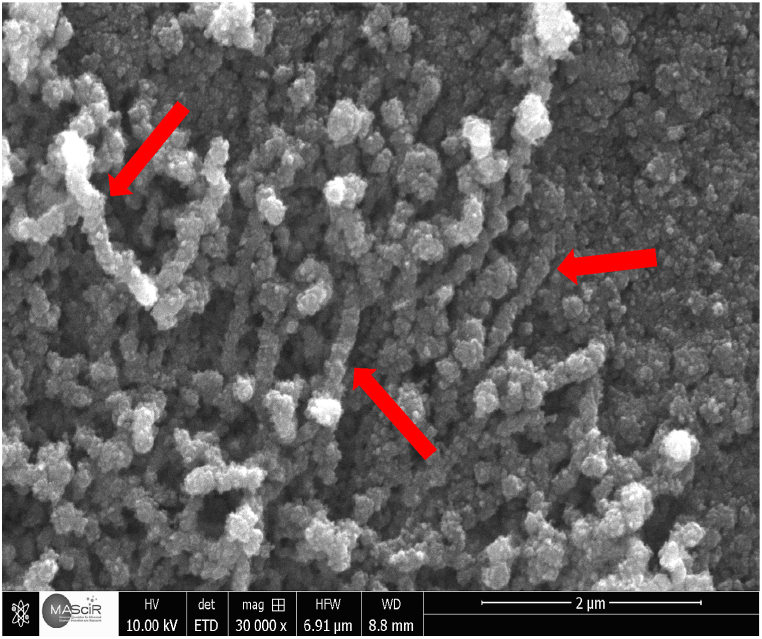
Surface morphology prepared after surface functionalization at 3 °C using toluene and HCl.

Moreover, even the choice of solvent and the choice of acid has an effect on the morphology of the coating, when xylene and HNO_3_ were changed to toluene and HCl, it was observed that at 3 °C the formation of nanofibers instead of raspberry nanoparticles (Fig. 5).

**Fig. 6:**
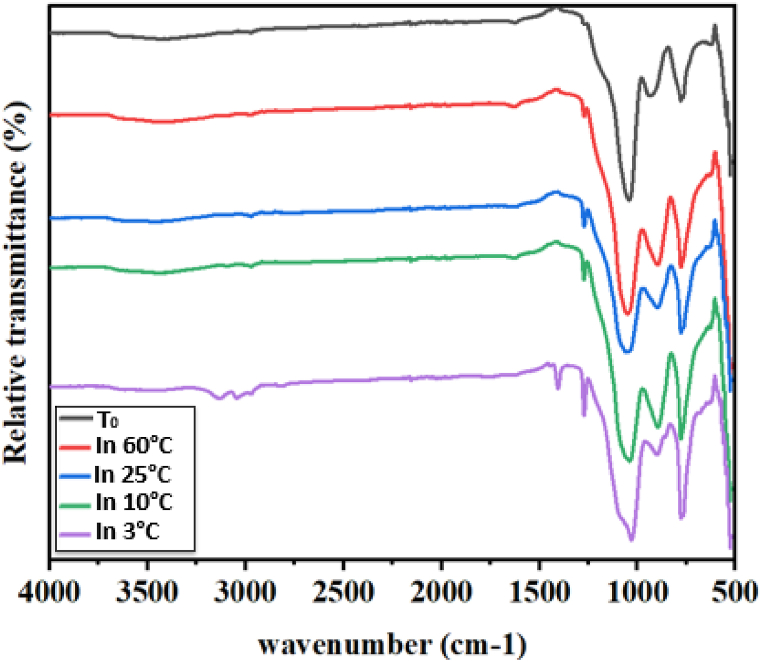
Infrared spectrum of the surfaces before the functionalization step named T0 and after the functionalization at different temperatures.

In order to investigate this matter, infrared analysis was carried out on multiple substrates prepared using the same method and in the four temperatures 60 °C, 25 °C, 10 °C, and 3 °C. The findings are summarized in Fig. 6. Certain peaks were observed at 1100 cm^−1^, 470 cm^−1^, and 781 cm^−1^, which correspond to the symmetrical stretching and bending vibrations of Si–O–Si. This confirms the formation of a three-dimensional network of SiO_2_ or the occurrence of a functionalization reaction between TCMS and SiO_2_ nanoparticles, leading to the creation of Si–O–Si bonds. Moreover, the absorption peak at 950 cm^−1^ and the prominent peak at 3394 cm^−1^ are associated with the stretching vibrations of –OH and Si–OH, respectively, indicating the presence of unmodified surface OH groups. Upon surface modification with MTCS, the intensity of –OH and Si–OH stretching vibrations decreased, while two new peaks appeared in the modified curve at 3000 cm^−1^ and 1275 cm^−1^. These peaks correspond to the vibrations of C–H and Si–CH_3_ bonds, respectively, suggesting the initial replacement of OH groups on the silica nanoparticle surface with grafted CH_3_ groups, as stated by Zhong et al. [36]. However, it is important to note that the intensity of these two peaks varies with the reaction temperature. They become more pronounced at lower temperatures during the functionalization reaction, while their intensity weakens at higher temperatures. This indicates that at high temperatures, surface functionalization primarily occurs through grafting reactions, resulting in less intense CH_3_ peaks. On the other hand, at lower temperatures, a polymerization reaction takes place within the reaction medium, leading to more intense CH_3_ peaks. It should be emphasized that these tests have been repeated three times, consistently yielding the same results.

### 3.2 Polymerization time effect

**Fig. 7:**
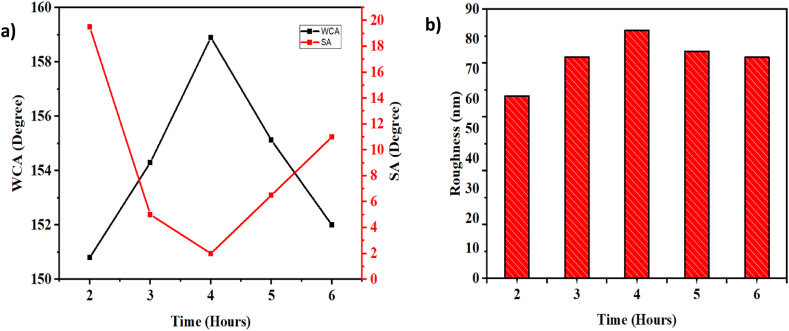
a) The variation of the contact angle and the slip angle as a function of functionalization time. b) The variation of the roughness as a function of the functionalization time.

After examining the impact of temperature on surface wettability during functionalization reactions, we will now explore the influence of functionalization time on surface properties in this section. To do so, we have maintained the SiO_2_ concentration at 1.5 % and the functionalization reaction temperature at 3 °C, while varying the polymerization time from 2 to 6 h. The results of this investigation are summarized in Fig. 7-a. Notably, we observed that the contact angle gradually increases with longer polymerization times, peaking at 158.9° after 4 h. Beyond the 4-h mark, the contact angle begins to decrease, reaching 155.12° and 152° for a functionalization time of 5 h and 6 h, respectively. Furthermore, the roughness values exhibit a consistent pattern corresponding to the contact angle values. The roughness increases as the polymerization time extends up to 4 h, reaching a maximum value of 83.01 nm before starting to decline (see Fig. 7-b).

**Fig. 8:**
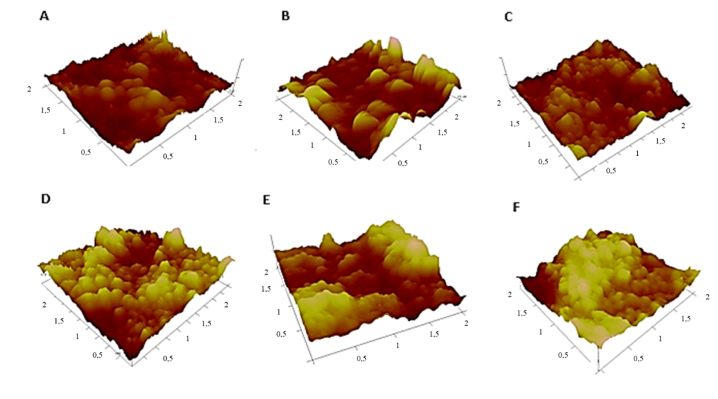
3D AFM profiles of surfaces as a function of functionalization time: A)-before functionnalization, B)- 2 h, C)- 3 h, D)- 4 h, E)- 5 h, F)- 6 h.

The surfaces were studied using AFM to shed light on this phenomenon. Fig. 8's 3D profiles show that the surface gradually gets more porous and hierarchical as the polymerization time rises, peaking at 4 h. When this period of time is exceeded, however, a definite structure in the shape of plateaus together with less defined but broader peaks starts to appear. In consequence, less trapped air between the macrostructures and a reduction in peak depth prevent water droplets from penetrating the surface. The reported values of roughness and contact angle are explained by this finding.

In reality, it is expected that when SiO_2_ nanoparticles are arranged, a hierarchical roughness in the form of peaks and cavities is formed (Fig. 8-A). The TCMS precursors will polymerize randomly on the surface of the nanoparticles after their addition, thus creating large raspberry-shaped nanoparticles that cover the entire surface and gradually increase the surface roughness (Fig. 8-D). However, as long as monomers are present in the reaction medium, the polymerization will continue. Consequently, after 4 h of reaction, the TCMS monomers will continue to polymerize, leading to an increasing formation of large nanoparticles that block the pores created by the SiO_2_ nanoparticles and the accumulation of raspberry nanoparticles on the surface forming plateaus (Fig. 8-F). This reduces the surface roughness and is consistent with the AFM findings.

### 3.3 Effect of SiO_2_ concentration

**Fig. 9:**
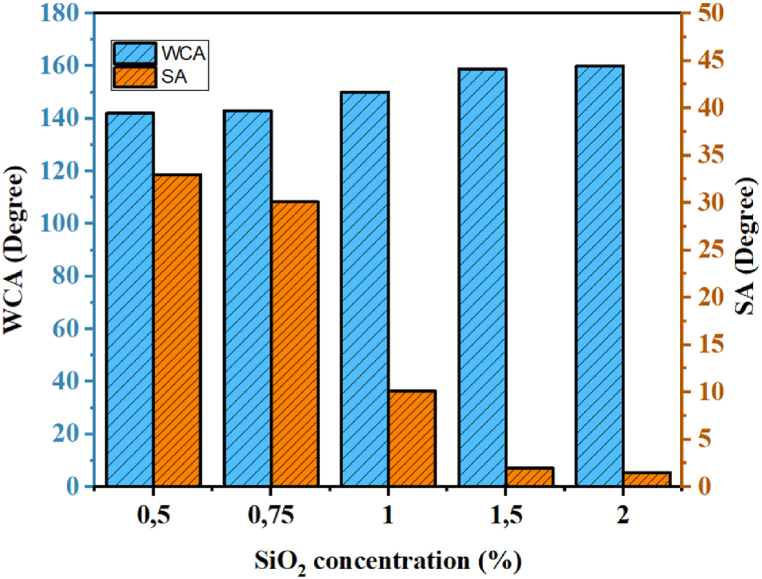
The variation of contact angle and slip angle as a function of SiO2 concentration.

It is commonly accepted that variations in nanoparticle concentration have a direct influence on the contact angle between the surface and water. Indeed, nanoparticles play a major role in creating surface roughness. To study this effect, the concentration of SiO_2_ nanoparticles varied from 0.5 % to 2 %, while the functionalization time was kept constant at 4 h and the reaction temperature at 3 °C. The results of this study are presented in Fig. 9. It can be observed that an increase in SiO_2_ nanoparticle concentration leads to an increase in the contact angle with water and a decrease in the sliding angle. Superhydrophobicity, characterized by a contact angle of 150° and a sliding angle below 10°, was only achieved at a SiO_2_ concentration of 1 % and above. At a concentration of 2 %, the surface exhibited a maximum contact angle with water of 158.9°, with a sliding angle close to 1.5°. The variation in concentration was halted at 2 % as it was observed that concentrations higher than 2 % result in a loss of transparency. This phenomenon is mainly attributed to the effect of surface roughness. As the deposition concentration of SiO_2_ increases, a hierarchical morphology forms, characterized by deeper pores capable of trapping a larger surface area between their nanostructures. This prevents water droplet penetration and consequently leads to an increase in the surface contact angle.

For the remaining part of this study, we will maintain a constant surface functionalization time of 4 h, while conducting the functionalization reaction at a temperature of 3 °C. These two factors have shown the most promising antiwetting outcomes. Additionally, we will examine the impact of SiO_2_ concentration on other properties.

### 3.4 Durability of superhydrophobic coating

#### 3.4.1 Thermal stability

**Fig. 10:**
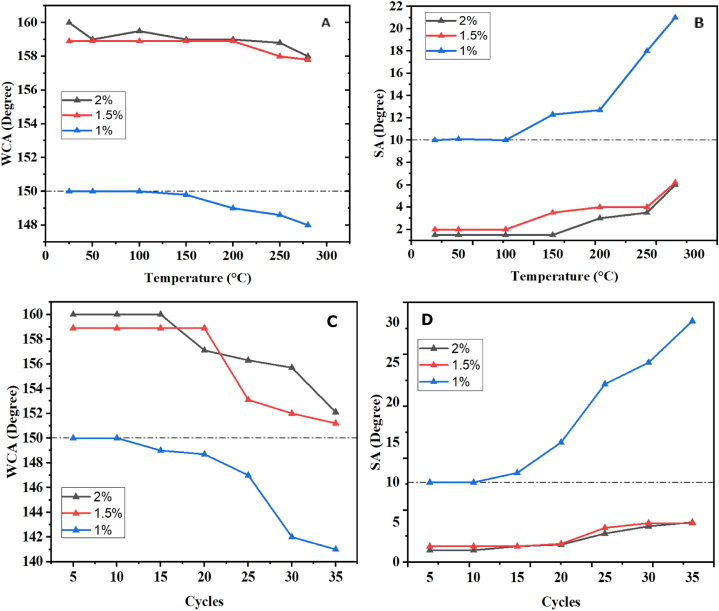
A)- The variation of the contact angle as a function of the temperature. B)- The variation of the sliding angle as a function of the temperature. C)- The variation of the contact angle as a function of the abrasion cycles. D)- The variation of the sliding angle as a function of the abrasion cycles.

To assess the thermal durability of the coatings, the coated substrates were subjected to various temperatures ranging from 25 °C to 280 °C for 1 h each. Fig. 10-A and Fig. 10-B presents the contact angle and sliding angle measurements as a function of temperature variation for different SiO_2_ concentrations. Remarkably, coatings with concentrations of 2 % and 1.5 % demonstrated exceptional thermal resistance, even at 280 °C, without any loss of superhydrophobicity or transparency. This remarkable resistance can be attributed to two primary factors. Firstly, the SiO_2_ nanoparticles exhibited excellent thermal stability within the temperature range of 25 °C–280 °C, maintaining their structural integrity without undergoing thermal decomposition. Secondly, the utilization of a silica-based resin, prepared through the sol-gel method, contributed to the coatings' ability to withstand high temperatures without decomposition, owing to the presence of robust Si–O–Si bonds. This distinguishes them from certain organic resins commonly employed in transparent and superhydrophobic coating preparations, which tend to decompose at specific temperatures and consequently lose their superhydrophobic properties. This sets it apart from several organic resins that are frequently utilized in the creation of coatings that are transparent and superhydrophobic. These resins lose their superhydrophobic qualities when they break down at particular temperatures. For instance, Kumar et al. [37] created a polymethyl methacrylate-based superhydrophobic coating that held up well in heat testing up to 170 °C. Similar to this, Aditya et al. [38] created a superhydrophobic coating using polyurethane that likewise resisted heat testing up to 175 °C. But after a while, neither covering retained its superhydrophobic properties.

#### 3.4.2 Abrasion test

It is crucial to ensure the mechanical stability of superhydrophobic coatings for their validation on an industrial scale [39]. To assess this, we subjected glass samples to mechanical damage by rubbing them with 200 g sandpaper over a distance of 10 cm per cycle. Fig. 10-C and Fig. 10-D presents the variations in contact angle and sliding angle as a function of the number of cycles for each SiO_2_ concentration.

**Fig. 11:**
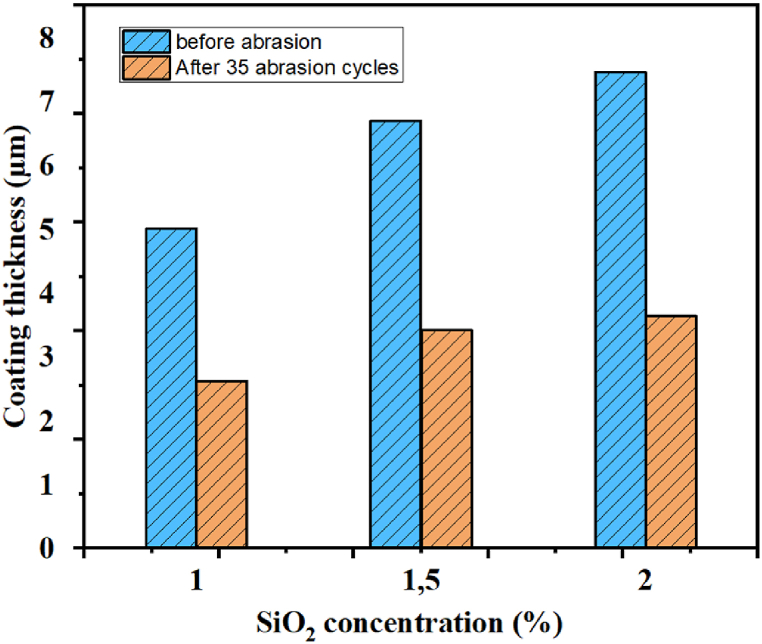
The variation in the thickness of the coating before and after the abrasion cycles.

The results revealed that reducing the SiO_2_ concentration can compromise the coating's resistance to abrasion. Specifically, samples with concentrations of 2 % and 1.5 % maintained their superhydrophobicity even after 35 cycles of abrasion. The contact angles decreased from 160° to 158.9°–152.7° and 151.2°, respectively, while the sliding angle remained below 5°. On the other hand, surfaces with low SiO_2_ concentrations rapidly lost their superhydrophobicity, as indicated by contact angles approaching 150°, resulting in the deterioration of their superhydrophobic properties over time. The contact angle decrease of the three concentrations can be attributed to two factors. Firstly, the loss of surface structure and subsequent reduction in surface roughness caused by mechanical action. Secondly, the film thickness decreases as the number of abrasion cycles increases, as shown in Fig. 11.

In comparison to coatings developed in the literature, they demonstrate durability levels that are either comparable or inferior in durability tests, such as those reported by Wenjing et al. [5] (20 cycles), Chong et al. [13] (20 cycles), Momen et al. [40] (20 cycles), or Sebastian et al. [41] (20 cycles).

#### 3.4.3 UV resistance

**Fig. 12:**
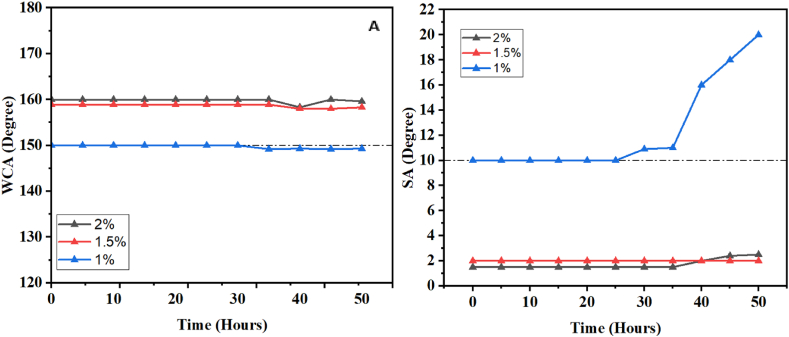
A)- The variation of the contact angle as a function of the UV irradiation time. B)- The variation of the sliding angle as a function of the UV irradiation time.

Solar energy, lenses, and glazing are applications that require the use of materials highly resistant to UV radiation in order to ensure a long lifespan after exposure to the outdoors. To accomplish this, we attempted to assess the UV resistance of our material. The coated substrates are placed in a machine where they undergo accelerated UV irradiation for 50 h, and the contact and sliding angles are measured every 5 h, as shown in Fig. 12. It was observed that increasing the concentration of SiO_2_ can enhance the surface's resistance to UV radiation. Samples with a higher SiO_2_ concentration exhibit excellent resistance, as they were able to maintain their superhydrophobicity even after 150 h at nearly 150 °C, with little to no change in the contact angle. Conversely, samples prepared with a 1 % SiO_2_ concentration lost their superhydrophobicity after 30 h. This demonstrates that increasing the SiO_2_ content can improve the UV resistance of our coating.

#### 3.4.4 Chemical stability

**Fig. 13:**
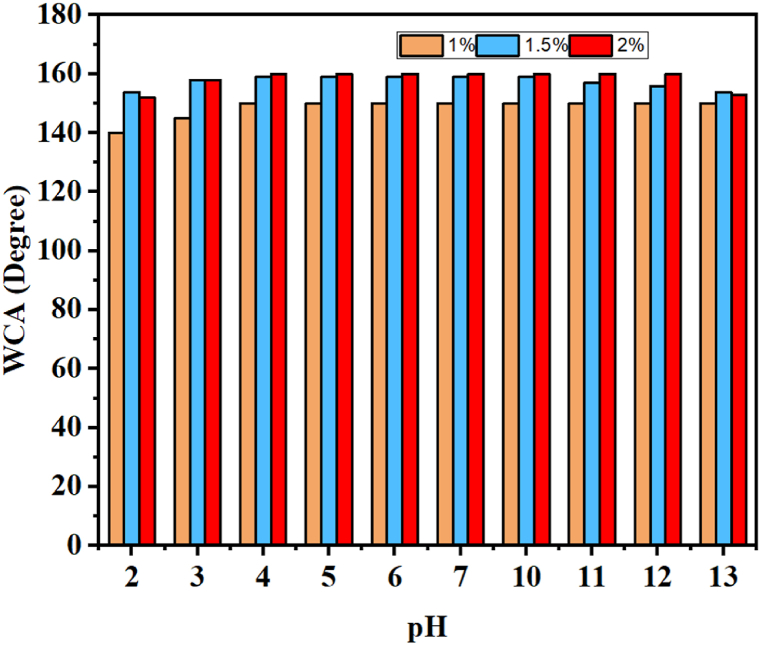
The variation of the contact angle as a function of the pH of solution in which the coatings have been immersed for 3 days.

To assess the chemical stability of the coatings, the coated substrates were submerged in NaOH and HCl solutions of varying pH levels (ranging from 2 to 13) for a duration of 3 days. Contact angle measurements were performed for the different samples and grouped in Fig. 13. Remarkably, it was observed that the pH of the solutions had no impact on the surface's ability to resist wetting, even after being immersed in highly aggressive environments for an extended period. This indicates the remarkable chemical stability of the coating. These results demonstrate the excellent chemical stability of our coating compared to others recently published in the literature, such as those by Zou et al. [42] and Li et al. [43], who prepared transparent and superhydrophobic coatings with chemical resistance not exceeding 80 min and 200 min, respectively.

#### 3.4.5 Sand abrasion

**Fig. 14:**
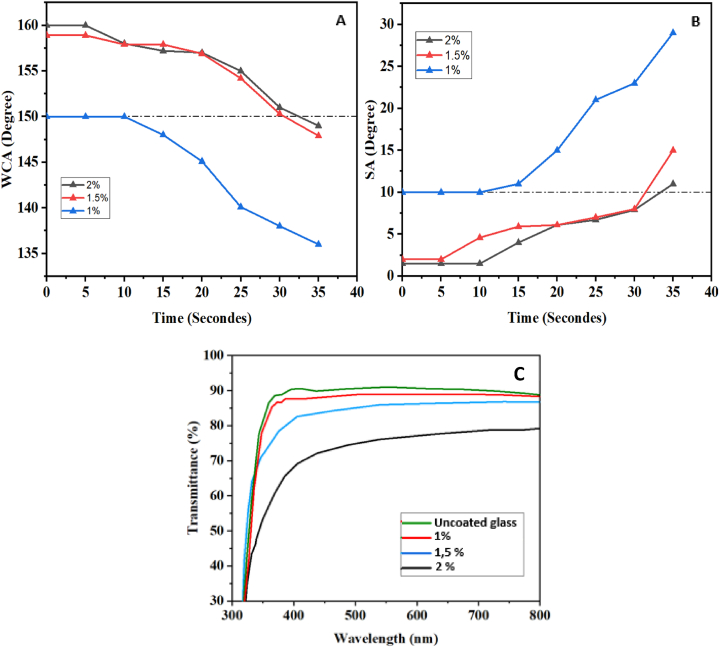
A)- The variation of the contact angle as a function of the sand abrasion time. B)- The variation of the sliding angle as a function of the sand abrasion time. C)- The transmittance of the samples as a function of the concentration of SiO2 used.

A sizable amount of sand with a diameter of 250 μm was made available close to a location where solar panels would be installed for the sand abrasion test. The sand was put into a pistol that can project sand depending on the applied pressure. This firearm was created in our lab to investigate the impact of erosion on surfaces under accelerated or realistic settings. The substrates that were coated with a layer were set up at a 90° angle and placed a good 80 cm away from the spraying apparatus. The substrates were subjected to a pressure of 6 bars, which is 9 times greater than the typical pressure in our area. The values of the contact angle and sliding angle were assessed at intervals of 5 s, and the gathered information is shown in Fig. 14. According to the findings, our coatings, which were created with concentrations of 1.5 % and 2 %, shown exceptional resilience against sand abrasion. These coatings retained their superhydrophobic qualities even after 30 s of bombardment, in contrast to substrates with lowest SiO_2_ contents, which started to lose them before the 30-s mark. This finding demonstrates how little sand abrasion affects our coating, making it appropriate for industrial applications in a variety of industries that demand erosion resistance.

### 3.5 Study of transparency

The use of a self-cleaning coating necessitates the critical combination of exceptional transparency (transmittance >80 %) and superhydrophobicity for industrial acceptability in a number of industries, including solar energy, building glazing, and optical device manufacture. Increasing the required surface roughness for superhydrophobicity results in a decrease in transparency, and vice versa, making superhydrophobicity and transparency two opposing qualities. Controlling and enhancing surface roughness is crucial for balancing these two qualities. To reduce Mie scattering, this roughness should ideally be lower than 100 nm, or less than one-fourth of the visible light wavelength. Based on the concentration of SiO_2_ nanoparticles, Fig. 14-C shows the percentage of sample transmission. Regardless of the quantity of SiO_2_ utilized, it can be shown that our technology enabled us to build superhydrophobic surfaces with better optical qualities to fulfill industrial needs. Additionally, it was shown that increasing the concentration of nanoparticles causes a drop in transmittance, which was anticipated given that an increase in SiO_2_ causes an increase in roughness. This demonstrates once more how conflicting roughness and transparency are as characteristics.

For a 1 % SiO_2_ concentration, it is possible to achieve a contact angle greater than 150° with a transmittance exceeding 90 %, representing a mere 2 % loss compared to uncoated glass. The difference is minimal for the 1.5 % and 2 % concentrations, which showed maximum contact angles of 158.9° and 160°, respectively. The 1.5 % concentration sample has 82 % transmittance, which is much greater than the 2 % concentration's transmittance of less than 80 %, which may not be suitable for all applications.

**Fig. 15:**
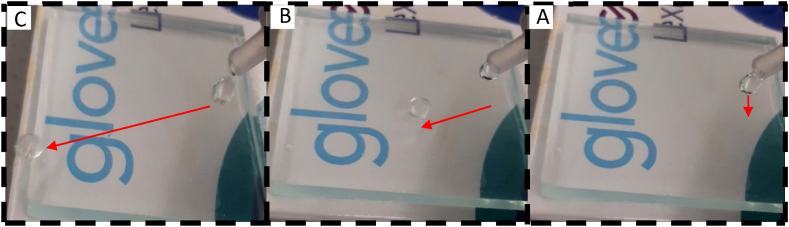
A photo of a transparent surface prepared with 1.5 %. A)- The drop of water falls on the superhydrophobic surface inclined with an angle of 0°, B)- the water drop quickly slides on the surface with high speed, C)- the drop of water jumps when leaving the substrate.

Therefore, a 1.5 % concentration of SiO_2_ nanoparticles may be the optimal concentration to generate a mix of very water-repellent and ultra-transparent surfaces. After our coating was applied to the substrates at a 1.5 % concentration, Fig. 15 shows how transparent they were. When looking at what is behind them, it is evident that the coating has no effect on the substrates' transparency.

Compared to other works, our coating has shown better or similar transparency, such as the study by Shang et al. [44], who fabricated a superhydrophobic coating with raspberry nanoparticles showing a transmittance of 82 %. Another study by Hao et al. [45] prepared a coating based on silica nanoparticles in combination with two polyurethane resins, showing a transmittance of less than 80 %.

### 3.6 Self-cleaning application

The adoption of water droplets in a spherical form and their ease of sliding on the surface make it self-cleaning, enabling the spontaneous and easy removal of dust, and even preventing its deposition on the surface. This self-cleaning property is highly sought after in various industrial applications. Based on the study of optimizing superhydrophobicity, transparency, and durability, it has been found that a concentration of 1.5 % SiO_2_, combined with surface functionalization for 4 h at a temperature of 3 °C, is the best combination for preparing transparent and mechanically and chemically stable superhydrophobic surfaces. Therefore, we conducted tests to evaluate the self-cleaning ability of this surface using two different methods.

**Fig. 16:**
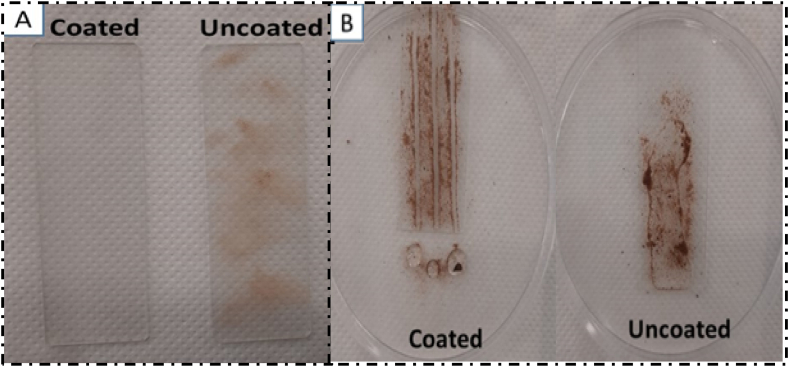
A) -the difference between coated and uncoated substrates from outdoor exposure self-cleaning test for 8 h. B) - the difference between coated and uncoated substrates from indoor sand deposition test.

In the first phase, the coated and uncoated surfaces were exposed outdoors for 8 h in a desert region and inclined at the same angle. Fig. 16-A shows the photos of the coated and uncoated substrates after their recovery. It can be observed that the coated surface remained perfectly clean, while a significant amount of dust was deposited on the uncoated surface, making the glass dirty. This dust accumulation leads to a 37 % decrease in substrate transmittance, demonstrating the effect of the coating in preventing dust deposition on the glass surface. This is due to the presence of the CH_3_ group on the surface, which prevents the formation of bonds with dust particles, thus preventing their adhesion to the surface.

The second self-cleaning test applied to our superhydrophobic coating involved spreading sand on both surfaces and then draining the water droplets (Fig. 16-B). When the water droplet fell on the uncoated surface, it was observed to spread across the surface due to its hydrophilic nature, without any cleaning effect. However, when the droplet was deposited on the coated surface, it formed a spherical shape and quickly slid down the surface under gravity, carrying away the dust particles. This resulted in a clean area, demonstrating the effect of the coating in facilitating the self-cleaning process. A quantity of 10 mg of dust was deposited on the coated substrate, and then a single droplet was applied to the substrate. The remaining dust on the surface was weighed again, revealing that a single droplet of 0.1 mL of water was capable of removing 6.07 mg of dust, thanks to the self-cleaning properties of the coating.

**Fig. 17:**
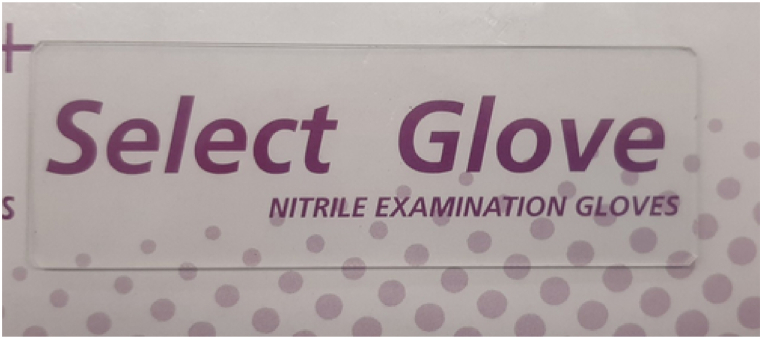
Substrate transparency after dust removal.

Furthermore, after depositing a sufficient quantity of droplets to clean the entire surface, it was observed that the transparency of the coating before dust deposition was practically the same as after dust cleaning (Fig. 17). This shows that all particles are eliminated, regardless of their size or shape. On the other hand, it was found that 0.15 mL was more than enough to clean the entire surface of the coated glass, while the uncoated surface required 2.5 mL for cleaning. This demonstrates that the coating prevents dust deposition on the surface, facilitates cleaning, and optimizes the amount of water needed for surface cleaning, reducing water consumption by up to 16 times compared to the uncoated surface. This can be a significant advantage for large surfaces and solar panel installations. Additionally, after cleaning the glass with water and allowing it to dry, stains would form on the surface. However, thanks to the use of superhydrophobic coating and the rapid sliding of droplets off the surface, this issue is significantly resolved.

## 4 Conclusion

In this study, we introduced a novel approach to fabricate a thermally controllable superhydrophobic and highly transparent surface with raspbery nanoparticleself-generated dual-scale roughness, eliminating the need for two different-sized nanoparticles. The impact of nanoparticle concentration, functionalization time, and reaction temperature on surface properties was investigated. Our findings revealed that a 1.5 % concentration of SiO_2_ nanoparticles, coupled with a 4-h TCMS surface functionalization at 3 °C, yielded the optimal conditions for achieving a surface that combines superhydrophobicity, transparency, and durability. This surface exhibited a contact angle of 158.8°, a sliding angle of 2°, and a transmittance of 83 %.

Moreover, the coating demonstrated exceptional stability against abrasion, enduring up to 35 cycles, and exhibited resistance to temperatures as high as 280 °C. It also displayed improved resistance to UV radiation for up to 50 h and enhanced protection against sand abrasion for up to 30 s under a bombardment pressure of up to 6 bars. Additionally, the surface demonstrated remarkable self-cleaning capabilities helps prevent dust from depositing on the surface, facilitates cleaning, and minimizes the amount of water used for cleaning.

## CRediT authorship contribution statement

**Brahim Nomeir:** Writing – review & editing, Writing – original draft, Methodology, Investigation, Formal analysis, Conceptualization. **Sara Lakhouil:** Writing – review & editing, Writing – original draft, Methodology, Investigation. **Sanae Naamane:** Validation, Supervision, Project administration, Funding acquisition, Conceptualization. **Mustapha Ait Ali:** Validation, Supervision. **Sofia Boukheir:** Validation, Supervision, Project administration, Funding acquisition.

## Declaration of competing interest

The authors declare that they have no known competing financial interests or personal relationships that could have appeared to influence the work reported in this paper.
