# Supplementary material for: Durable, transparent and superhydrophobic coating with temperature-controlled dual-scale roughness by self-assembled raspberry nanoparticles
Source: Heliyon. 2024 Jul 23;10(15):e34983. doi: 10.1016/j.heliyon.2024.e34983 (PMC11336374; doi:10.1016/j.heliyon.2024.e34983)
Supplement: Multimedia component 1 [file mmc1.docx]

**Durable, transparent and superhydrophobic coating with temperature-controlled dual-scale roughness by self-assembled raspberry nanoparticles**

Brahim Nomeir^1,2^, Sara Lakhouil^2,3^, Sanae Naamane^2^, Mustapha Ait Ali^1^,Sofia Boukheir^2^

^1^Molecular Chemistry Laboratory, Unit Coordination and Catalysis Chemistry, Cadi Ayyad University, Faculty of Sciences Semlalia (UCA-FSSM), B.P. 2390 – 40000 Marrakech, Morocco

^2^Moroccan foundation for Advanced Science Innovation and Research (MAScIR), Rue Mohamed Al Jazouli, Rabat, Morocco

^3^Mohammed V University, Mohammadia School of Engineers (EMI), Avenue Ibn Sina B.P. 765 Agdal, Rabat, Morocco

a) **Corresponding author:** [b.nomeir@mascir.ma](mailto:b.nomeir@mascir.ma)

b)

a)


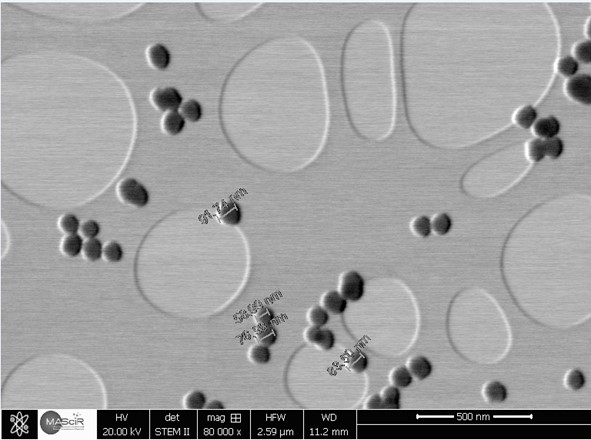

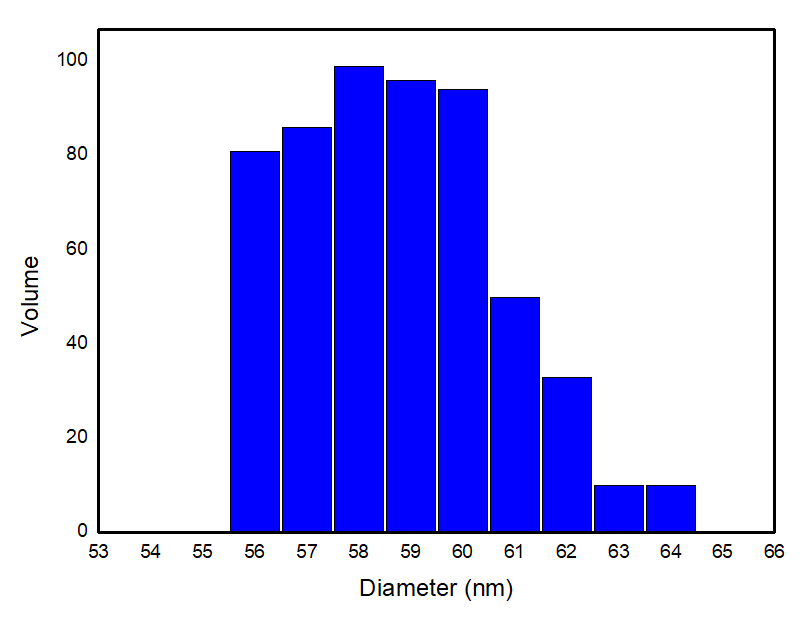


Figure 1: characterization of the nanoparticles size by a) Zetasizer and b) SEM.

**Standard deviation calculation:**

For each sample, we performed five measurements per sample for each metric and each durability test. The error bars were calculated as the standard deviation of these measurements using the following method: first, we calculated the mean (average) value for each set of measurements; next, we subtracted the mean from each individual measurement to find the deviation of each measurement from the mean; then, we squared each of these deviations; after that, we calculated the average of these squared deviations; finally, we took the square root of this average to obtain the standard deviation. The number of replicates performed for each measurement is specified in the figure captions.

- **The impact of temperature on surface morphology and wettability**

Table 1 The variation of the WCA contact angle and roughness as a function of temperature variations during the functionalization reaction.

| Temperature (°c) | Contact angle (°) | Roughness (nm) |
| --- | --- | --- |
| 3 | 158,9±1,8 | 83,3±3,2 |
| 10 | 154,3±3 | 72,2±1,3 |
| 25 | 152,1±2,1 | 67,9±2,1 |
| 60 | 151±2,8 | 62,1±3,5 |

- **Polymerization time effect**

Table 2: The variation of the contact angle, slip angle and roughness as a function of functionalization time.

| Time (hours) | Contact angle (°) | Sliding angle (°) | Roughness (nm) |
| --- | --- | --- | --- |
| 2 | 150,7 ±3,1 | 19,9±1,2 | 58,9±2,2 |
| 3 | 154,3± 2,3 | 5,1±3,3 | 72,4± 2,1 |
| 4 | 158.9±1,5 | 2±1,9 | 83.01±2,3 |
| 5 | 155.12±1,4 | 6,2±3,2 | 72,6±4,1 |
| 6 | 152±2,4 | 11±2,2 | 71,1±1,2 |

**- Effect of SiO_2_ concentration**

Table 3: The variation of contact angle and slip angle as a function of SiO_2_ concentration.

| Nanoparticle concentration (%) | Contact angle (°) | Sliding angle (°) |
| --- | --- | --- |
| 0,5 | 141,5±2,22 | 32,5±1,2 |
| 0,75 | 142,4±1,3 | 30,1±3,4 |
| 1 | 151,6±2 | 10±3,1 |
| 1,5 | 158,9±1,09 | 2,7±2,1 |
| 2 | 159,9±2,01 | 2±1 |

**Durability of superhydrophobic coating:**

- **Thermal stability**

Table 4: The variation of the contact angle as a function of the temperature.

| Temperature (C°) | 1 | 1,5% | 2% |
| --- | --- | --- | --- |
| 25 | 151,6±2,1 | 158,9±1,3 | 159,9±3,5 |
| 50 | 151,6±1,2 | 158,9±2,2 | 158,9±1,43 |
| 100 | 151,3±2,01 | 158 ,9±3,5 | 159,1±1,22 |
| 150 | 149,2±3,3 | 158,9±2,3 | 158,9±2,01 |
| 200 | 149±1,9 | 158,9±2,11 | 158,9±1,92 |
| 250 | 148,3±2,2 | 158±2,9 | 158,9±3,5 |
| 280 | 148±3,5 | 157,9±2,2 | 157,9± 3,3 |

Table 5: The variation of the sliding angle as a function of the temperature.

| Concentrations (%) | 25 | 50 | 100 | 150 | 200 | 250 | 280 |
| --- | --- | --- | --- | --- | --- | --- | --- |
| 2 | 1,5±3,1 | 1,5±2,1 | 1,5±1,1 | 1,5±1,2 | 3±1,2 | 3,5±1,9 | 6±2,2 |
| 1,5 | 2±2,2 | 2±1,1 | 2±2,1 | 3,5±1,4 | 4±1,1 | 4±2,3 | 6,2±2,1 |
| 1 | 10±3,1 | 10,1±2,4 | 10±1,1 | 12,3±2,2 | 12,7±2,2 | 18±3,3 | 21±1,3 |

- **Abrasion test**

Table 6: The variation of the contact angle as a function of the abrasion cycles.

| Concentrations (%) | 5 | 10 | 15 | 20 | 25 | 30 | 35 |
| --- | --- | --- | --- | --- | --- | --- | --- |
| 2 | 160±1,45 | 160±2,12 | 160± 2 | 157,1±1 | 156,3±1,1 | 155,7±1 | 152,1±1 |
| 1,5 | 158,9±3,2 | 158,9±1,7 | 158,9±1 | 158,9±2,12 | 153,1±1,5 | 152±1,5 | 151,2±1 |
| 1 | 150±2,71 | 150± 2,31 | 149±1,4 | 148,7±2,1 | 147±1,53 | 142±1,4 | 141±1 |

Table 7: The variation of the sliding angle as a function of the abrasion cycles.

| Concentrations (%) | 5 | 10 | 15 | 20 | 25 | 30 | 35 |
| --- | --- | --- | --- | --- | --- | --- | --- |
| 2 | 1,5±3,2 | 1,5±1,45 | 2±2,1 | 2,2±2,2 | 3,6±2,3 | 4,5±1,2 | 5±1,2 |
| 1,5 | 2±1,8 | 2±2,21 | 2±3,3 | 2,3± 2,1 | 4,3±2,7 | 4,9±2,12 | 4,9±1,5 |
| 1 | 10±1,2 | 10±2,32 | 11,2±3,1 | 15±2,7 | 22,3±1,1 | 25±2,31 | 30,2±1,7 |

- **UV Resistance**

Table 8: The variation of the contact angle as a function of the UV irradiation time.

| Concentrations (%) | 5 | 10 | 15 | 20 | 25 | 30 | 35 | 40 | 45 | 50 |
| --- | --- | --- | --- | --- | --- | --- | --- | --- | --- | --- |
| 2 | 160±1,2 | 160±1,8 | 160± 1,1 | 160±1,2 | 160±1,7 | 160±1,45 | 160±3,1 | 158,3±2,31 | 160±1,61 | 159,6±1,21 |
| 1,5 | 158,9±3,2 | 158,9±3,1 | 158,9±1,21 | 158,9±1,11 | 158,9±1,44 | 158,9±1,51 | 158,9±3,1 | 158±1,45 | 158±2,33 | 158,3±1,6 |
| 1 | 150±2,1 | 150±1,1 | 150±3,4 | 150±1,23 | 150±1,71 | 150±1,1 | 149,2±3,13 | 149,3±3,12 | 149,2±2,1 | 149,3±1,8 |

Table 9: The variation of the sliding angle as a function of the UV irradiation time.

| Concentrations (%) | 5 | 10 | 15 | 20 | 25 | 30 | 35 | 40 | 45 | 50 |
| --- | --- | --- | --- | --- | --- | --- | --- | --- | --- | --- |
| 2 | 1,5±2,12 | 1,5±1,1 | 1,5±3,13 | 1,5±1,2 | 1,5±3,12 | 1,5±3,2 | 1,5±1,1 | 2±2,12 | 2,4±1,9 | 2,5±2,91 |
| 1,5 | 2±2,13 | 2±3,12 | 2±3,1 | 2±1,1 | 2±3,2 | 2±3,11 | 2±2,16 | 2±2,19 | 2±1,12 | 2± 2,3 |
| 1 | 10±3,7 | 10±2,1 | 10±1,1 | 10±2,1 | 10±3,12 | 10,9±3,21 | 11±2,1 | 16±1 | 18±2,4 | 20±3,2 |

- **Chemical stability**

Table 10: The variation of the contact angle as a function of the pH of solution in which the coatings have been immersed for 3 days.

| Concentrations (%) | 2 | 3 | 4 | 5 | 6 | 7 | 10 | 11 | 12 | 13 |
| --- | --- | --- | --- | --- | --- | --- | --- | --- | --- | --- |
| 2 | 152±3,1 | 158±3,12 | 160±1,55 | 160±1,11 | 160±2,89 | 160±2,4 | 160±1,1 | 160±1,4 | 160±2,1 | 153±1,4 |
| 1,5 | 153,9±1,1 | 157,9±3,14 | 158,9±1,51 | 158,9±1,51 | 158,9±3 | 158,9±1,77 | 158,9±2,1 | 156,9±2,1 | 155,9±1,3 | 153,9±1,2 |
| 1 | 140±1,5 | 145±1,09 | 150±1,15 | 150±1 | 150±1,21 | 150±4,9 | 150±2,1 | 150±1,32 | 150±1,3 | 150±2,1 |

- **Sand abrasion**

Table 11: The variation of the contact angle as a function of the sand abrasion time.

| Concentrations (%) | 5 | 10 | 15 | 20 | 25 | 30 | 35 |
| --- | --- | --- | --- | --- | --- | --- | --- |
| 2 | 160±3,1 | 158±1,2 | 157,2±1,3 | 157±1,2 | 155±2,1 | 151±2,1 | 149±3,33 |
| 1,5 | 158,9±2,2 | 157,9±2,3 | 157,9±2,2 | 156,9±1,3 | 154,2±1,2 | 150,3±1,2 | 147,9±1,3 |
| 1 | 150±4,1 | 150±1,2 | 148±3,1 | 145,1±1,31 | 140,1±2,1 | 138±2,16 | 136±1,6 |

Table 12: The variation of the sliding angle as a function of the sand abrasion time.

| Concentrations (%) | 5 | 10 | 15 | 20 | 25 | 30 | 35 |
| --- | --- | --- | --- | --- | --- | --- | --- |
| 2 | 1,5±0,5 | 1,5±3,31 | 4±1,21 | 6,1±2,22 | 6,7±1,55 | 7,9±0,4 | 11±3,2 |
| 1,5 | 2±2,19 | 4,6±1,34 | 5,9±2,55 | 6,1±1,43 | 7±1 | 8± 0,33 | 15±3,4 |
| 1 | 10±2,2 | 10±2,22 | 11±2,45 | 15±1,65 | 21±2,3 | 23±1,23 | 29±1,3 |
